# Periconceptional bread intakes indicate New Zealand's proposed mandatory folic acid fortification program may be outdated: results from a postpartum survey

**DOI:** 10.1186/1471-2393-12-8

**Published:** 2012-02-14

**Authors:** Simonette R Mallard, Andrew R Gray, Lisa A Houghton

**Affiliations:** 1 Department of Human Nutrition, University of Otago, PO Box 56, Dunedin 9054, New Zealand; 2 Department of Preventive and Social Medicine, University of Otago, PO Box 9013, Dunedin 9054, New Zealand

## Abstract

**Background:**

In September 2009, a folic acid fortification mandate (135 μg/100 g bread) was to be implemented in New Zealand. However, due to political and manufacturer objection, fortification was deferred until May 2012. Based on estimates of bread consumption derived from a 1997 nationally representative survey, this program was intended to deliver a mean additional intake of 140 μg folic acid/d to women of childbearing age. Little is known about current bread consumption patterns in this target group. The aim of this study was to assess bread consumption among women prior to and during pregnancy with the intent to estimate periconceptional folic acid intakes that would be derived from bread if mandatory fortification were implemented as currently proposed.

**Methods:**

A retrospective survey of 723 postpartum women in hospitals and birthing centres across New Zealand was conducted using a self-administered questionnaire on bread intake prior to and during pregnancy and maternal socio-demographic and obstetric characteristics.

**Results:**

Median bread intake before conception (2 slices/d) was below that of previous data upon which the current fortification proposal was modeled (3-4 slices/d). If mandatory fortification is implemented as proposed, only 31% (95% CI = 24%-37%) of childbearing-age women would attain an additional folic acid intake of ≥ 140 μg/d, with a mean of 119 μg/d (95% CI = 107 μg/d-130 μg/d). Based on these data, a fortification level of 160 μg/100 g bread is required to achieve the targeted mean of 140 μg folic acid/d. Nonetheless, under the current proposal additional folic acid intakes would be greatest among the least advantaged segments of the target population: Pacific and indigenous Māori ethnic groups; those with increased parity, lower income and education; younger and single mothers; and women with unplanned pregnancies. Subgroups predicted to derive less than adequate folic acid intakes from the proposed policy were women of Asian descent and those with a postgraduate education.

**Conclusions:**

This study provides insight on the ability of a fortification policy to benefit the groups at highest risk of poor folate intakes in a population. However, bread consumption among the target group of childbearing women appears to have declined since the data used in previous dietary modeling were collected. Thus, it seems prudent to re-model dietary folic acid intakes based on more recent national survey data prior to the implementation of a mandatory folic acid fortification policy.

## Background

Neural tube defects (NTD), including spina bifida, anencephaly and encephalocele, result from incomplete neural tube closure during early embryogenesis [1]. In addition to causing considerable perinatal mortality worldwide, NTD also give rise to infantile morbidity that often persists into adulthood [1]. Of all congenital malformations, NTD are among the most costly to treat, however they are unique in that more than two-thirds of cases are preventable by adequate intake of folic acid before and during the first trimester of pregnancy [1-3]. Although accurate estimates of the total incidence of NTD are unavailable for New Zealand, the approximate rate at birth is seven cases per 10,000 [4]. This rate is 40% higher than the lowest NTD birth prevalence estimated to be attainable with sufficient folic acid intake [5].

While mandatory fortification interventions provide a lower dose of folic acid than the issued supplement recommendation of 400 μg/d, existing programs have proven successful in both raising reproductive-aged women's red cell folate and reducing the incidence of NTD [6-9]. Moreover, mandatory folic acid fortification of a staple food delivers folic acid to those who may not otherwise commence folic acid supplementation preconceptionally, such as women with unplanned pregnancies [10]. In New Zealand, voluntary folic acid fortification of specified foods has been permitted since 1996, with breakfast cereals and wholegrain breads making up the majority of currently fortified food items [11]. The addition of folic acid to all yeast-leavened, non-organic bread was to be mandated in September 2009 at a level of 135 μg folic acid/100 g bread [11]. Using nationally representative data from a food consumption survey conducted in 1997, this fortification level was selected with the aim of achieving optimal effectiveness and safety [11]. The 1997 survey reported a daily median bread intake of 3-4 slices among reproductive-aged women, and the fortification level was intended to increase mean folic acid intakes of this group by 140 μg/d [11]. However, due to political and manufacturer objection, mandatory fortification was deferred until May 2012, with no guarantee of implementation [12].

Although bread was the chosen vehicle for fortification in New Zealand, little is known about the current bread consumption patterns of childbearing women prior to and during pregnancy. Moreover, no previous studies have determined the potential impact of fortification on folic acid intakes by age, socioeconomic status and ethnicity to evaluate whether the program would have a meaningful impact on all segments of the target population. This is particularly important given socio-demographic disparities in periconceptional folic acid supplement use reported in New Zealand [10], Australia [13], and elsewhere [14-16]. Therefore, we assessed bread consumption among women prior to and during pregnancy with the intent to estimate periconceptional folic acid intakes that would be derived from bread if mandatory fortification were implemented as currently proposed.

## Methods

### Study design and population

The Vitamins and Minerals in Pregnancy Survey was a retrospective survey of postpartum women in birthing centers and hospitals located across New Zealand between March 7 and April 15, 2011. Participating sites included: Tauranga Hospital, Tauranga; Whakatane Hospital, Whakatane; River Ridge East Birth Centre, Waterford Birth Centre and Waikato Hospital, Hamilton; Hutt Hospital, Lower Hutt; Kenepuru Community Hospital and Wellington Hospital, Wellington; Christchurch Women's Hospital, Christchurch; Queen Mary Maternity Hospital, Dunedin; and Southland Hospital, Invercargill. Women aged 18 y or over who had delivered a healthy term (≥ 37 wk gestation) infant and could communicate in English were eligible for participation. Ethical approval for this study was obtained from the Multi-Region Ethics Committee of the New Zealand Ministry of Health.

### Survey questionnaire

An anonymous, self-administered 65-item questionnaire was developed based on similar postpartum questionnaires used elsewhere [17-19]. The questionnaire included items on dietary supplement use and usual bread intake before and during pregnancy, as well as maternal socio-demographic and obstetric characteristics. Participants were asked whether they ate non-organic, shop-bought bread. If yes, participants were asked how many servings of bread they consumed per day in the month before pregnancy and during each trimester of pregnancy (i.e. months 1-3, 4-6 and 7-9 of pregnancy). One slice of bread with a median weight of 33.0 g [20] was defined as single serving, with items such as rolls, buns, pita bread, focaccia, Turkish pide, naan, bagels, English muffins and sweet buns being equivalent to two bread slices/servings. Using the proposed level of mandatory bread fortification of 135 μg folic acid per 100 g bread [11], the average daily folic acid intake from bread for each individual was estimated. Pre-testing and refinement of the questionnaire was undertaken in January 2011 with eight pregnant volunteers.

### Statistical analysis

Women were assigned to one of five mutually exclusive ethnic groups using the 2006 national Census question. Participants who nominated two or more ethnic groups were assigned to a single ethnic group using the prioritization system recommended by Statistics New Zealand, with the order of priority being (from highest to lowest): Māori, Pacific, Asian, Other and New Zealand European [21]. Proportional to recent national maternity data, under-represented age-ethnicity subgroups were weighted up and over-represented subgroups were weighted down to ensure estimates of intake were representative in terms of age and ethnicity [22]. All analyses incorporated post-stratification weights and included sites as clusters to estimate robust standard errors. Differences in bread consumption and folic acid intake derived from bread were calculated and the means of these estimated to examine within-person changes in intake between preconception and the first trimester. Linear regression analyses were performed to estimate mean intakes and 95% CI of folic acid derived from bread if fortified as proposed, in the month prior to conception and the first trimester of pregnancy. Independent variables included: maternal age (three categories), parity (four categories), ethnicity (five categories), education (five categories), household income (five categories), relationship status (living with a partner versus not), and pregnancy intention (planned versus unplanned). The level of fortification required to achieve the targeted mean additional folic acid intake of 140 μg per day was then estimated based on bread consumption prior to conception. All analyses were conducted using Stata 11.1 (Stata Corporation 2010, College Station, Texas, United States), and a two-sided 0.05 level of significance was used in all cases.

## Results

### Study sample characteristics

Of the 968 women invited to participate, 758 agreed. Thirty-five women did not meet inclusion criteria for maternal age or gestational duration, resulting in a total sample of 723 (75%). Median maternal age (IQR) was 31 y (8 y), similar to the national median recorded in the year ended March 2011 (30 y) [23]. Almost half of all deliveries were to primiparous women (45%) and 44% of all pregnancies were unplanned, comparable to data reported recently from a large, Auckland-based study (43% and 40%, respectively) [24]. Two-thirds (66%) of women held a post-secondary qualification, and 42% reported an annual household income below the national median for 2010 (64,272 NZ$) [25]. Most women were married or cohabitating (91%). Compared to recent national maternity data, this sample had a higher proportion of New Zealand Europeans (65% versus 56%), a lower proportion of Māori (14% versus 21%) and Pacific women (5% versus 11%), and a similar proportion of Asians (9% versus 10%) [22]. Post-stratification weighting was performed using age and ethnicity groups and analyses reported hereafter are weighted and incorporate robust standard errors with sites treated as clusters.

### Bread consumption in the periconceptional period

Before conception and during the first trimester of pregnancy 86% of women consumed bread that would be fortified if mandatory fortification were implemented as currently proposed. Mean bread intake in first trimester was greater than that prior to conception (+0.21 slices/d, 95% CI = 0.09-0.34; *p *= 0.004) (Table 1). Correspondingly, estimated mean folic acid intake derived from bread if fortified at the proposed level increased in the first trimester by almost 10 μg/d (9.53 μg/d, 95% CI = 3.92-15.16; *p *= 0.004).

**Table 1:** Daily bread and folic acid intake if fortification mandated as proposed prior to and during the first trimester^1^

Bread and derived folic acid intake	Mean (95% CI)^2^		Percentiles^2^				
			
			5	25	50	75	95
**Before conception, n 677**							

Bread intake, slices/d	2.6	(2.4-2.9)	0.0	2.0	2.0	4.0	6.0

Folic acid intake, μg/d^3^	118.5	(107.1-129.8)	0.0	89.1	89.1	178.2	267.3

**First trimester, n 676**							

Bread intake, slices/d	2.9	(2.5-3.2)	0.0	2.0	2.0	4.0	6.0

Folic acid intake, μg/d^3^	128.1	(112.2-144.0)	0.0	89.1	89.1	178.2	267.3

^1^135 μg folic acid/100 g bread

^2^Weighted by age and ethnicity and adjusted for clustering by site

^3^Median bread slice 33.0 g containing 44.6 μg folic acid/slice [20]

### Estimated folic acid intakes derived from bread, if fortified at the proposed level

Prior to conception, mean folic acid intake derived from bread if fortified as proposed increased linearly with parity and decreased linearly with increasing age, education and household income (Table 2). Pairwise comparisons revealed that Māori women would attain a higher intake of folic acid than New Zealand European women, Asian women, and women of Other ethnicity (all *p *≤ 0.001). Pacific women would attain a folic acid intake greater than that of Asian women and women of Other ethnicity (*p *= 0.048 and *p *= 0.021, respectively). Asian women born in New Zealand (n = 6) would attain a mean folic acid intake of 126 μg/d compared to 85 μg/d among Asian women born outside of New Zealand (n = 50) (*p = *0.27, data not shown). On average, women reporting an unplanned pregnancy would achieve a folic acid intake 38 μg in excess of that among women reporting a planned pregnancy, while women not living with a partner would achieve a folic acid intake 62 μg in excess of married/cohabitating women.

**Table 2:** Maternal factors and estimated periconceptional daily intake of folic acid, if fortification mandated as proposed^1^

	Folic acid intake from bread before conception^2^				Folic acid intake from bread in the first trimester^2^			
**Maternal characteristic**	**n**	**μg/d**	***P-*value**	**< 140 μg/d**	**n**	**μg/d**	***P-*value**	**< 140 μg/d**

		*Mean (95% CI)*		*% (95% CI)*		*Mean (95% CI)*		*% (95% CI)*

**All respondents**	677	118.5 (107.1-129.8)	< 0.001	69.3 (62.9-75.7)	676	128.1 (112.2-144.0)	< 0.001	65.1 (59.7-70.5)

**Age at delivery**			0.001^L^				0.001^L^	

< 25	103	144.5 (130.7-158.3)		58.1 (50.3-66.0)	103	155.0 (126.2-183.8)		57.4 (48.3-66.5)

25-34	390	116.1 (101.7-130.5)		70.9 (62.6-79.2)	390	127.7 (105.6-149.7)		65.3 (58.0-72.6)

> 35	181	108.9 (95.4-122.4)		72.2 (63.8-80.5)	180	114.2 (97.4-131.0)		69.0 (60.5-77.4)

**Parity**			0.006^L^				0.022^L^	

Primiparous	305	108.9 (93.1-124.7)		72.3 (65.3-80.6)	305	119.0 (98.9-139.0)		68.0 (59.4-76.7)

2 children	197	109.0 (95.8-122.2)		75.8 (69.7-82.0)	195	125.0 (97.4-152.6)		70.1 (65.3-74.9)

3 children	116	131.9 (115.9-147.9)		62.2 (54.1-70.2)	117	131.2 (113.6-148.8)		60.5 (53.8-67.2)

≥ 4 children	57	162.9 (128.3-197.6)		49.0 (30.3-67.7)	57	168.2 (135.6-200.9)		49.7 (32.5-67.0)

**Prioritised ethnicity**			0.015				0.111	

Māori	93	173.1 (144.8-201.5)		47.1 (35.6-58.7)	91	191.2 (139.4-243.0)		49.0 (39.0-59.1)

Pacific	35	142.7 (102.5-182.8)		57.7 (44.5-70.8)	35	144.5 (86.1-202.9)		55.5 (40.3-70.7)

Asian	56	88.5 (59.0-118.0)		81.8 (72.1-91.5)	56	95.4 (56.6-134.2)		78.7 (66.8-90.6)

NZ European	428	103.4 (96.8-109.9)		76.0 (70.9-81.1)	429	113.3 (103.5-123.1)		69.0 (64.4-73.6)

Other	57	97.4 (87.2-107.6)		80.3 (75.4-85.3)	57	100.9 (88.4-113.5)		78.6 (73.3-84.0)

**Highest education qualification**			0.002^L^				0.002^L^	

Less than high school	92	169.0 (131.6-206.3)		48.3 (34.2-62.5)	92	196.5 (137.6-255.5)		46.7 (34.8-58.6)

High school	132	126.7 (106.2-147.3)		64.0 (51.1-76.8)	132	129.8 (107.6-152.0)		60.5 (47.6-73.3)

Vocational training	56	121.1 (93.9-148.2)		69.0 (53.6-84.5)	57	144.7 (115.9-173.4)		59.1 (42.6-75.6)

Tertiary qualification	318	107.7 (97.0-118.4)		74.7 (70.0-79.4)	317	113.6 (103.0-124.2)		69.6 (64.0-75.1)

Postgraduate qualification	70	83.3 (57.3-109.4)		84.6 (72.7-96.4)	70	87.0 (56.1-118.0)		82.3 (69.3-95.4)

**Annual household income, NZ$**			0.007^L^				0.011^L^	

< 40,000	157	139.2 (105.3-173.1)		60.4 (48.4-72.4)	158	162.3 (112.8-211.9)		56.7 (46.0-67.3)

41,000-60,000	112	122.9 (114.3-131.6)		68.8 (62.7-74.8)	111	125.8 (115.3-136.3)		66.2 (60.3-72.1)

61,000-80,000	103	118.7 (99.5-137.9)		67.3 (53.5-81.2)	104	126.3 (107.3-145.2)		61.4 (48.3-74.4)

81,000-100,000	107	102.9 (86.2-119.6)		73.1 (62.5-83.7)	107	105.0 (93.9-116.2)		70.1 (63.3-77.0)

> 100,000	169	98.5 (85.3-111.6)		80.2 (73.7-86.6)	168	108.7 (87.4-130.0)		72.7 (61.0-84.5)

**Relationship status**			0.015				0.019	

Not living with a partner	64	174.4 (126.3-222.5)		49.9 (29.3-70.6)	64	185.9 (132.7-239.1)		49.4 (33.3-65.5)

Married/cohabitating	611	112.4 (103.7-121.0)		71.4 (66.2-76.6)	610	121.8 (108.2-135.4)		66.7 (62.2-71.3)

**Pregnancy intention**			0.001				0.026	

Unplanned	289	139.5 (122.9-156.1)		60.6 (51.7-69.5)	290	145.4 (124.4-166.4)		56.9 (50.3-63.6)

Planned	383	101.3 (88.7-113.9)		76.5 (71.4-81.6)	381	113.3 (92.7-133.8)		72.3 (66.5-78.2)

^1^135 μg folic acid/100 g bread

^2^Weighted by age and ethnicity and adjusted for clustering by site. NZ, New Zealand. ^L^*P*-value for linear trend

Should bread be fortified at the proposed level, more than two-thirds of women would fail to achieve the intended folic acid intake of 140 μg/d in the period before conception. Over 80% of women of Asian and Other ethnicity, women with a postgraduate qualification and women in the highest household income bracket would not attain a preconceptional folic acid intake of 140 μg/d if bread were fortified as currently proposed. In contrast, more than half of Māori women, women not living with a partner, women without a high school education and women with four or more children would reach a folic acid intake of ≥ 140 μg/d preconceptionally under the current fortification proposal. In the first trimester, patterns of folic acid intake derived from bread if fortified as proposed remained similar to before conception, although Pacific women would no longer attain a significantly higher intake than women of Asian or Other ethnicity.

**Figure 1:**
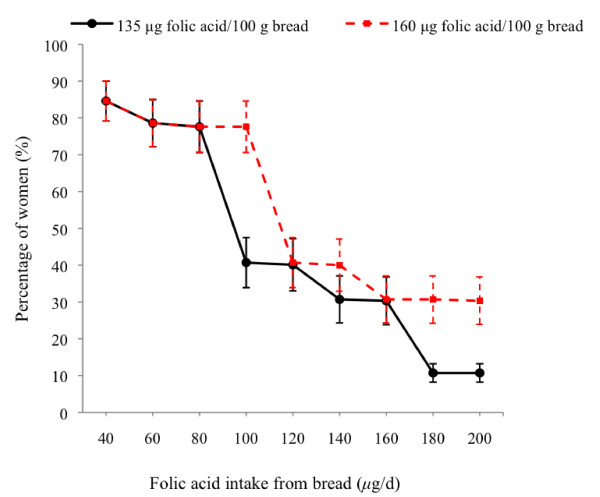
**Proportion of women attaining specified intakes of folic acid from bread alone**. Folic acid intakes estimated from reported bread consumption before conception among postpartum women. Solid line represents if bread fortification mandated as proposed at 135 μg folic acid/100 g bread, and dashed line if bread fortification mandated at 160 μg folic acid/100 g bread. Values are % (95% CI), weighted by age and ethnicity and adjusted for clustering by site, n = 677.

To achieve an average intake of 140 μg of folic acid/d from bread among childbearing women, we calculate that a fortification level of 160 μg folic acid/100 g bread is required (Figure 1). At this fortification level, 78% of all women would attain a folic acid intake derived from bread of ≥ 100 μg/d, with a median folic acid intake of 106 μg/d. In comparison, if bread were fortified as currently proposed, only 41% of all women would attain a folic acid intake derived from bread of ≥ 100 μg/d, with a median folic acid intake of 89 μg/d.

## Discussion

Nearly 15 y have elapsed since the collection of data upon which New Zealand's current mandatory fortification proposal is based, and our data indicates bread consumption has decreased markedly from a median of 3-4 slices/d to 2 slices/d in the target group of childbearing-age women. If mandatory fortification is implemented as proposed, we estimate the age- and ethnicity-weighted mean additional folic acid intake to be 119 μg/d among women prior to pregnancy, well below the intended 140 μg/d. Given the bread consumption data collected in this study, to achieve the targeted additional intake of 140 μg/d, a mandatory fortification level of 160 μg folic acid/100 g bread may be required. Nonetheless, the results of our study indicate that the least advantaged segments of the target population will benefit from the mandate if implemented as currently proposed. Additional folic acid intakes were shown to be greatest among Pacific and indigenous Māori ethnic groups, those with increased parity, lower income and education, younger and single mothers and women with unplanned pregnancies. Conversely, subgroups predicted to derive the least benefit from the proposed bread scheme are women of Asian descent and those with a postgraduate education.

Despite a non-binding agreement from the bread industry in 2009 to increase the number of voluntarily fortified breads, few breads in New Zealand contain added folic acid [26]. Fortified breads are typically wholegrain, which may not be readily affordable for women of lower socioeconomic status [26]. In the 2008/2009 Adult Nutrition Survey, women living in the most deprived neighborhoods were less likely to regularly consume wholegrain bread than those in the least deprived neighborhoods, and the overall proportion of women regularly consuming wholegrain bread declined linearly with decreasing age [27]. The other main source of dietary folic acid in New Zealand is voluntarily fortified ready-to-eat breakfast cereals [11]. In the 1997 National Nutrition Survey, those living in areas of greatest socioeconomic deprivation and Māori and Pacific people consumed the least servings of cereals (including breakfast cereals) per week [28]. Disparity in the consumption of voluntarily fortified foods is also apparent in current estimates of dietary folic acid intake. From voluntarily fortified sources, the mean intake of folic acid among New Zealand women is estimated to be 58 μg/d. Comparatively, the median intake is estimated to be 21 μg/d, suggesting that many women consume close to nil folic acid from voluntarily fortified foods [11]. Here, we are able to show that the proposed bread fortification mandate would remedy these identified socio-demographic inequities in dietary folic acid intake, with young women, Māori and Pacific women and those with lower household incomes benefiting markedly from the proposed scheme. Recommended periconceptional folic acid supplement use is also lower among these groups, ranging from 2.7% among Pacific women, 9.2% among Maori women, and 7% among women reporting an annual household income of less than 40,000 NZ$ [10]. This further underscores the benefit of mandatory fortification in reducing inequities in folic acid intake. To our knowledge, no published data on the rate of NTD among different ethnic groups in New Zealand exists post-introduction of periconceptional folic acid supplementation recommendations. However, reports from the late 1970's and early 1980's indicate that the rate of NTD was lower among Pacific and Māori women compared with non-Māori [29].

Although fortification is shown to be effective at reaching a wide range of individuals, it should be highlighted that 14% of all women either consumed no bread in the periconceptional period, or consumed only organic or homemade bread, which are exempt under the current proposal. Where all enriched cereal-grain products are fortified in the US at 140 μg folic acid/100 g flour, bread has been proposed as the solitary food vehicle for mandatory fortification in New Zealand [11,30]. While having greater tractability for monitoring purposes, bread fortification will not benefit those who consume little or no bread, and will likely result in lower folic acid intakes than if all cereal-grain products were fortified. In the US, a median folic acid intake of 117 μg/d was reported among adult women whose folic acid intakes were exclusively derived through the consumption of mandatorily fortified cereal-grain products [30]. Our estimated median additional intake of 89 μg folic acid/d among New Zealand women prior to conception is substantially lower. At a higher fortification level of 160 μg folic acid/100 g bread, we predict a median additional intake of 106 μg/d, closer to that of US women.

In addition to bread, mandatory folic acid fortification of further food products may be needed to target specific population subgroups. For example, Asian women were estimated to attain a mean additional folic acid intake 30 μg/d below the overall mean, indicating that this subgroup is targeted less effectively under the proposed program. Acculturation may influence bread consumption [31], as Asian women born outside of New Zealand consumed approximately one slice of bread less per day than those born in New Zealand. A recent Auckland-based study found that Asian women also consumed fewer servings of ready-to-eat breakfast cereal than New Zealand Europeans, placing this group at further risk of poor folic acid intakes [32]. Other potential cereal-grain fortification vehicles, such as rice and noodle products, are predominantly imported into New Zealand and are therefore not readily amenable to mandatory fortification. Fortification of eggs is a possible alternative [33], with egg intake among Asian women reported to be significantly higher than among New Zealand Europeans [32]. Compared to New Zealand Europeans, periconceptional folic acid supplement use is also lower among Asian women [10], further identifying this group as being at high-risk of suboptimal folic acid intakes.

Internationally, mandatory folic acid fortification interventions are based on country-specific target intakes, risk-assessment and consumption patterns, and range from 140-220 μg folic acid/100 g cereal-grain product [34]. Our proposed fortification level of 160 μg folic acid/100 g bread to achieve a targeted mean additional intake of 140 μg/d in childbearing-age women is therefore not unreasonable, although dietary modeling for the entire population would be necessary to ensure folic acid intakes remain within acceptable levels. In Australia, folic acid intakes from voluntarily fortified foods are estimated to be higher than those in New Zealand, and mandatory folic acid fortification of bread (200 μg per 100 g flour, equivalent to 120 μg per 100 g bread) was introduced in September 2009 [11]. Mean red cell folate levels measured in a large sample of Australians undergoing diagnostic blood tests increased from 881 nmol/L prior to mandatory fortification to 1071 nmol/L in the year ended April 2010 [35]. In a randomised controlled trial designed to mimic intended folic acid intakes of the proposed fortification program in New Zealand, participating reproductive-aged women had a baseline mean red cell folate level of 753 nmol/L [36]. Following 40 weeks folic acid supplementation at 140 μg/d, mean red cell folate levels rose to 1111 nmol/L [36]. These findings show that mandatory folic acid fortification in Australia at the current level and in New Zealand at its intended intake level (140 μg/d) is successful in raising red cell folate levels to those associated with the maximal reduction in NTD risk (906 nmol/L) [37].

Several limitations in our study merit discussion, such as its retrospective design with reliance on memory of past bread intake. Moreover, we did not formally validate our survey questions for assessing the habitual bread consumption over the defined periods of time. Underreporting of energy intakes occurs more frequently among non-pregnant women than men, and underreporting of energy intakes in pregnancy has recently been correlated with socio-demographic status [38]. Underreporting of bread consumption may thus have lead to an underestimation of folic acid intakes, which may have occurred differentially according to socio-demographic subgroup, potentially attenuating or exacerbating estimated differences in folic acid intake between groups. However, the anonymous, self-administered nature of the survey and its completion prior to discharge may have lessened under-reporting and incorrect recall, respectively. In addition, retrospective nutrient intakes in pregnancy have been reported elsewhere to have a validity similar to those conducted in the general populace [39]. While a lower proportion of Māori and Pacific women participated than would be expected given national data, this was due to the non-inclusion of hospitals in the Northland region, where a large proportion of people identifying themselves as Māori and Pacific reside [40,41]. Weighting by age and ethnicity resolved these differences in ethnic representation, thus there are no reasons to expect this to have affected our estimation of folic acid intakes.

## Conclusions

Mandatory fortification of enriched cereal-grain products in the US has been highly effective in reducing the incidence of NTD [8]. In two large US case-control studies, it has recently been found that supplemental folic acid does not confer any further benefit in the prevention of NTD, suggesting an optimum habitual intake has been reached [42,43]. In New Zealand, mandatory bread fortification would deliver substantial additional folic acid intakes to socio-demographic subgroups of women where consumption of voluntarily fortified foods and folic acid supplement uptake during the periconceptional period is insufficient. As many countries worldwide continue to debate mandatory folic acid fortification, this study provides insight on the ability of such a policy to benefit the groups at highest risk of poor folate intakes in a population. Nonetheless, in light of the finding that not all subgroups may benefit equally, this study underscores the importance of monitoring actual folate intakes and status in the target population should fortification be mandated. Bread consumption among reproductive-aged women appears to have declined since the data used in previous dietary modeling were collected. It would therefore be prudent to re-model population-wide folic acid intakes using more recent national survey data to assess the impact of the proposed fortification policy prior to its implementation.

## Competing interests

The authors declare that they have no competing interests.

## Authors' contributions

SRM designed the study and the data collection tools, monitored the data collection, planned analyses, cleaned and analysed the data and drafted the paper. ARG contributed to study design and planning of analyses, analysed the data and revised the draft paper. LAH conceived the idea for the study, was involved in study design, monitored data collection, revised the draft paper and is guarantor. All authors have read and approved the final version of the manuscript.

## Pre-publication history

The pre-publication history for this paper can be accessed here:

http://www.biomedcentral.com/1471-2393/12/8/prepub
